# A Population-Based Analysis of Diabetes-Related Care Measures, Foot Complications, and Amputation During the COVID-19 Pandemic in Ontario, Canada

**DOI:** 10.1001/jamanetworkopen.2021.42354

**Published:** 2022-01-05

**Authors:** Charles de Mestral, David Gomez, Andrew S. Wilton, Douglas S. Lee, Zaina Albalawi, Peter C. Austin, Jean Jacob-Brassard, David R. Urbach, Mohammed Al-Omran, Nancy N. Baxter

**Affiliations:** 1 Li Ka Shing Knowledge Institute of St Michael’s Hospital, Toronto, Ontario, Canada; 2 ICES, Toronto, Ontario, Canada; 3 Department of Surgery, Temerty Faculty of Medicine, University of Toronto, Ontario, Canada; 4 Diabetes Action Canada, Toronto, Ontario, Canada; 5 Department of Medicine, Temerty Faculty of Medicine, University of Toronto, Ontario, Canada; 6 Division of Endocrinology and Metabolism, Faculty of Medicine, Memorial University, St John’s, Newfoundland and Labrador, Canada; 7 Melbourne School of Population and Global Health, University of Melbourne, Melbourne, Victoria, Australia

## Abstract

**Importance:**

Deferred diabetic foot screening and delays in timely care of acute foot complications during the COVID-19 pandemic may have contributed to an increase in limb loss.

**Objective:**

To evaluate the association of the COVID-19 pandemic with diabetes-related care measures, foot complications, and amputation.

**Design, Setting, and Participants:**

This population-based cohort study included all adult residents of Ontario, Canada, with diabetes and compared the rates of selected outcomes from January 1, 2020, to February 23, 2021, vs January 1, 2019, to February 23, 2020.

**Main Outcomes and Measures:**

Comprehensive in-person diabetes care assessment, including foot examination; hemoglobin A_1c_ (HbA_1c_) measurement; emergency department visit or hospitalization for diabetic foot ulceration, osteomyelitis, or gangrene; lower extremity open or endovascular revascularization; minor (toe or partial-foot) amputation; and major (above-ankle) leg amputation. Rates and rate ratios (RRs) comparing 2020-2021 vs 2019-2020 for each measure were calculated for 10-week periods, anchored relative to onset of the pandemic on March 11, 2020 (11th week of 2020).

**Results:**

On March 11, 2020, the study included 1 488 605 adults with diabetes (median [IQR] age, 65 [55-74] years; 776 665 [52.2%] men), and on March 11, 2019, the study included 1 441 029 adults with diabetes (median [IQR] age, 65 [55-74] years; 751 459 [52.1%] men). After the onset of the pandemic, rates of major amputation in 2020-2021 decreased compared with 2019-2020 levels. The RR for the prepandemic period from January 1 to March 10 was 1.05 (95% CI, 0.88-1.25), with RRs in the pandemic periods ranging from 0.86 (95% CI, 0.72-1.03) in May 20 to July 28 to 0.95 (95% CI, 0.80-1.13) in October 7 to December 15. There were no consistent differences in demographic characteristics or comorbidities of patients undergoing amputation in the 2020-2021 vs 2019-2020 periods. Rates of comprehensive in-person diabetes care assessment and HbA_1c_ measurement declined sharply and remained below 2019-2020 levels (eg, in-person assessment, March 11 to May 19: RR, 0.28; 95% CI, 0.28-0.28). The rates of emergency department visits (eg, March 11 to May 19: RR, 0.67; 95% CI, 0.61-0.75), hospitalization (eg, March 11 to May 19: RR, 0.77; 95% CI, 0.68-0.87), open revascularization (eg, March 11 to May 19: RR, 0.66; 95% CI, 0.56-0.79), endovascular revascularization (March 11 to May 19: RR, 0.70; 95% CI, 0.61-0.81), and minor amputation (March 11 to May 19: RR, 0.70; 95% CI, 0.60-0.83) initially dropped but recovered to 2019-2020 levels over the study period.

**Conclusions and Relevance:**

In this population-based cohort study, disruptions in care related to the COVID-19 pandemic were not associated with excess leg amputations among people living with diabetes. As the pandemic ends, improved prevention and treatment of diabetic foot complications will be necessary to maintain these positive results.

## Key Points

**Question:**

Were disruptions in diabetes-related care during the COVID-19 pandemic associated with a higher rate of foot complications and amputation in Ontario, Canada?

**Findings:**

In this cohort study of more than 1.4 million adults with diabetes, care measures, foot complications, and leg amputation during the first 2 waves of the COVID-19 pandemic were lower than prepandemic levels. There were no consistent differences in demographic characteristics and comorbidities among patients undergoing leg amputation during the pandemic compared with those who underwent amputation in 2019 to 2020.

**Meaning:**

In this study, despite limited ambulatory in-person assessment by physicians, hospital avoidance, and restrictions to scheduled hospital-based procedures, excess leg amputations were not observed among people living with diabetes during the first 11 months of the COVID-19 pandemic.

## Teaser

This cohort study evaluates the association of the COVID-19 pandemic with diabetes-related care measures, foot complications, and amputation among adults in Ontario, Canada.

## Introduction

Among people living with diabetes, leg amputation is a feared complication that negatively affects both quality and longevity of life. Frequently synergistic, loss of protective sensation from neuropathy and limited blood flow from peripheral artery disease heighten the risk of foot wounds, infection, and gangrene. Fortunately, limb loss may be prevented through guideline-aligned care including patient education, periodic foot screening, glycemia and cardiovascular risk factor management, and timely interdisciplinary treatment of acute foot complications.^1,2,3,4^

The COVID-19 pandemic has resulted in additional challenges for people living with diabetes that may be associated with the risk of foot ulcer development and amputation. Reports from Italy, China, India, and the United States are cause for concern with 2- to 10-fold increases in leg amputations noted.^5,6,7,8,9^ The pandemic may have contributed to a rise in diabetes-related amputations for a number of reasons. In-person diabetic foot screening, which should include foot examination, assessment of footwear, and interventions to prevent ulceration (eg, debridement of callus), was likely limited during periods of lockdown. Unprecedented emergency department (ED) avoidance during the pandemic may also have contributed to delayed presentation and a greater likelihood of progression to severe infection or extensive gangrene.^10^ Finally, relative restriction to scheduled invasive interventions, including revascularization, may have contributed to delays in correcting arterial insufficiency in people with diabetes and limb-threatening ischemia. On the other hand, 2 recent population-based studies of people with diabetes in England and France identified a decrease in limb loss during the pandemic, potentially explained by limited daily activities during lockdown translating to fewer neuropathic foot ulcers.^11,12^ However, these analyses did not capture the comorbidity profile of individuals undergoing amputation during the pandemic relative to historical controls to explore the possibility of excess mortality precluding amputation during the pandemic. Furthermore, diabetes care measures (eg, foot examination and hemoglobin A_1c_ [HbA_1c_] measurement) associated with short- and long-term amputation risk were not studied.

Data remain needed to allay or substantiate concerns about disruptions in care and resulting greater limb loss among people with diabetes since the onset of the COVID-19 pandemic. We therefore sought to quantify the association of the pandemic with diabetes-related care measures, foot complications, and leg amputation.

## Methods

### Study Design, Setting, and Ethical Approval

We conducted a population-based cohort study of adults with diabetes using linked administrative health data sets for the province of Ontario, Canada. For the nearly 14 million residents of Ontario, all hospital care, physician services, and investigations are funded within a single-payer public health care system. The use of data in this project was authorized under section 45 of Ontario’s Personal Health Information Protection Act, which does not require review by a research ethics board or informed consent from participants. The study reporting followed Strengthening the Reporting of Observational Studies in Epidemiology (STROBE) reporting guideline for cohort studies.

Among adults with diabetes, the rates of 8 diabetes care measures and outcomes, including major (ie, above-ankle) leg amputation, were considered. To understand changes in these rates through different phases of the pandemic, the cohort enrollment and observation period was segmented into six 10-week periods from January 1, 2020, to February 23, 2021, and 6 corresponding 10-week control periods from January 1, 2019, to February 23, 2020: January 1 to March 10 (weeks 1-10), March 11 to May 19 (weeks 11-20), May 20 to July 28 (weeks 21-30), July 29 to October 6 (weeks 31-40), October 7 to December 15 (weeks 41-50), and December 16 to February 23 (weeks 51-60).

Landmark dates informed the selection of the 10-week timeframe: March 11, 2020 (11th week of 2020), was the date of the first reported death from COVID-19 in the province of Ontario and the same day the World Health Organization declared a state of pandemic. By March 19, 2020, the provincial Ministry of Health had mandated a province-wide suspension of nonessential scheduled health services and, by May 19, 2020, a gradual resumption of scheduled procedures and in-person health services was permitted.^13^ Prior work has shown that, in addition to scheduled invasive procedures, a slowdown in urgent invasive procedures also occurred in Ontario within this period.^14,15,16^ On July 24, 2020, the provincial state of emergency enacted during the first wave was lifted.^13^ On September 28, 2020, the beginning of the second wave of COVID-19 was officially recognized by the provincial government.^17^ Indoor gatherings with people outside a person’s household were prohibited on December 26, 2021.^13^ A provincial state of emergency was reinstated from January 14 to February 19, 2021, during the latter half of the second wave.^13^

### Study Population and Data Sources

Data sets held at ICES served as the data sources (eAppendix 1 in the Supplement).^18^ ICES is an independent, nonprofit research institute whose legal status under Ontario’s health information privacy law allows it to collect and analyze health care and demographic data, without consent, for health system evaluation and improvement.

For each of the six 10-week periods in 2020-2021 and corresponding 10-week control periods in 2019-2020, all adult Ontario residents with type 1 or 2 diabetes alive at the beginning of the period were identified from the Registered Persons Database and the Ontario Diabetes Database*.* The occurrence of major amputation, related outcome and care measures as well as the demographic characteristics and comorbidities of adults with diabetes and those undergoing major amputation were captured using previously described or validated approaches from linked population-based data sets of all hospitalizations, emergency department visits, and surgeries as well as physician and laboratory service claims (eAppendix 1 in the Supplement). Death or loss of Ontario resident status was available from the Registered Persons Database. These data sets were linked using unique encoded identifiers and analyzed at ICES.

### Diabetes Care Measures, Foot Complications, and Leg Amputation

In addition to major (above-ankle) amputation, 7 related diabetes care and outcome measures were captured among people with diabetes: (1) comprehensive in-person diabetes care assessment, based on physician fee incentives that explicitly require foot examination, by primary care, endocrinology, or general internal medicine physicians; (2) HbA_1c_ measurement; (3) ED visit for diabetic foot ulceration, osteomyelitis, or gangrene; (4) hospitalization for a diabetic foot ulceration, osteomyelitis, or gangrene; (5) minor (toe or partial-foot) amputation; (6) endovascular lower extremity revascularization; and (7) open surgical lower extremity revascularization (eAppendix 1 in the Supplement). These diverse outcome and care measures capture efforts to prevent and treat foot complications of diabetes. Based on evidence-based practice guidelines and prior analyses, these measures can be expected to affect the short- and long-term risk of leg amputation among people with diabetes.^1,2,3,4,19,20,21^

### Characteristics of All Adults With Diabetes and Those Undergoing Amputation

Demographic characteristics and selected comorbidities of relevance to amputation risk were captured for all adults with diabetes and major amputation patients. These included age, sex, neighborhood income level, rural residence, Northern Ontario residence, living in a long-term care facility, congestive heart failure, hypertension, chronic obstructive pulmonary disease, coronary artery disease, chronic kidney disease, and peripheral artery disease (eAppendix 1 in the Supplement).

### Statistical Analysis

For each of the previously defined 10-week time periods, each person’s rate of a given event (eg, major amputations) was calculated as the number of events per weeks at risk. The observation window for a person’s total weeks at risk began at the start of the given 10-week time period (eg, January 1) and ended at (1) the 10-week mark (eg, March 10), (2) the time of death, or (3) the loss of Ontario resident status, whichever came first.

For each of the 6 periods in 2020-2021, a rate ratio (RR) and 95% CIs were then calculated relative to the corresponding period in 2019-2020, using generalized estimation equations accounting for within-participant correlation. The time period was the independent variable, and the number of events (eg, major amputations) was the dependent variable, assuming a negative binomial distribution, with an offset variable denoting the observation window.

To identify potential differences in the types of patients undergoing leg amputation during the pandemic vs prepandemic periods, the demographic characteristics and comorbidities of these patients were compared between 2020-2021 and 2019-2020 time periods. We used χ^2^ and Kruskal-Wallis tests for categorical and continuous variables, respectively.

In addition, to understand the longitudinal risk of major amputation without segmenting the observation period, a supplemental analysis was performed. Among the cohort of all adults with diabetes at the onset of the pandemic on March 11, 2020, the cumulative incidence of amputation, as well as death as a competing risk, was calculated during the complete 50-week follow-up period (March 11, 2020, to February 23, 2021), with censoring for end of follow-up without amputation or death. The same estimates over 50 weeks of follow-up were calculated for the control cohort of all adults with diabetes on March 11, 2019. All statistical tests were 2-sided, with statistical significance defined as *P* < .05. Analyses were performed using SAS version 9.5 (SAS Institute).

## Results

At the onset of the pandemic on March 11, 2020, there were a total of 1 488 605 adults with diabetes (median [IQR] age, 65 [55-74] years; 776 665 [52.2%] men), and their demographic characteristics and comorbidities were similar to those of the 1 441 029 adults with diabetes (median [IQR] age, 65 [55-74] years; 751 459 [52.1%] men) on the historical reference date of March 11, 2019 (Table 1). Across time intervals during the pandemic, there were no consistent differences in demographic characteristics or comorbidities between individuals undergoing major amputation in 2020-2021 vs 2019-2020 (Table 2).

**Table 1.** Demographic Characteristics and Comorbidities of Adults With Diabetes at Onset of Pandemic (March 11, 2020) and Control Date (March 11, 2019)

Covariates^a^	Patients, No. (%)	
	Mar 11, 2020 (n = 1 488 605)	Mar 11, 2019 (n = 1 441 029)
Age, median (IQR), y	65 (55-74)	65 (55-74)
Sex		
Male	776 665 (52.2)	751 459 (52.1)
Female	711 940 (47.8)	689 570 (47.9)
Rural residence^b^	155 375 (10.4)	151 371 (10.5)
Northern Ontario resident	94 334 (6.3)	92 677 (6.4)
Lowest income quintile^c^	346 378 (23.3)	338 312 (23.5)
Long-term care resident	37 410 (2.5)	36 831 (2.6)
Congestive heart failure	131 028 (8.8)	126 956 (8.8)
Hypertension	984 128 (66.1)	958 977 (66.5)
COPD	259 140 (17.3)	253 684 (17.4)
Coronary artery disease	91 058 (6.1)	91 609 (6.4)
Chronic kidney disease	72 157 (4.8)	68 066 (4.7)
Peripheral artery disease	19 880 (1.3)	19 451 (1.3)

Abbreviation: COPD, chronic obstructive pulmonary disease.

^a^ Standardized difference was 0.01 or less for all covariates.

^b^ Rural residence was missing for 6348 participants (0.4%) on March 11, 2020, and 5108 (0.4%) on March 11, 2019.

^c^ Income quintile missing for 7059 participants (0.5%) on March 11, 2020, and 5794 (0.4%) on March 11, 2019.

**Table 2.** Demographic Characteristics and Comorbidities of Individuals Undergoing Amputation in Each Time Period

Characteristic	Patients by time period, No. (%)											
	Pre–COVID-19		Wave 1				Between waves		Wave 2			
	Jan 1-Mar 10		Mar 11-May 19		May 20-Jul 28		Jul 29-Oct 6		Oct 7-Dec 15		Dec 16-Feb 23	
	2020 (n = 275)	2019 (n = 259)	2020 (n = 235)	2019 (n = 243)	2020 (n = 223)	2019 (n = 252)	2020 (n = 239)	2019 (n = 255-259)^a^	2020 (n = 257-261)^a^	2019 (n = 264)	2020-2021 (n = 241)	2019-2020 (n = 258)
Age, median (IQR), y	66 (58-73)	67 (57-76)	66 (57-74)	67 (58-76)	66 (58-73)	67 (58-75)	65 (55-73)^b^	68 (60-77)^b^	67 (59-76)	66 (58-75)	67 (59-75)	66 (58-74)
Sex												
Male	198 (72.0)	187 (72.2)	168 (71.5)	179 (73.7)	164 (73.5)	185 (73.4)	172 (72.0)	186-190 (72.9-73.4)	181-185 (70.4-70.9)	186 (70.5)	192 (79.7)	190 (73.6)
Female	77 (28.0)	72 (27.8)	67 (28.5)	64 (26.3)	59 (26.5)	67 (26.6)	67 (28.0)	69-73 (27.1-28.2)	76-80 (29.6-30.7)	78 (29.5)	49 (20.3)	68 (26.4)
Rural residence^c^	47 (17.1)	37 (14.3)	52 (22.1)	47 (19.3)	36 (16.1)	50 (19.8)	51 (21.3)	39-43 (15.3-16.6)	39-44 (15.2-16.9)	49 (18.6)	47 (19.5)	44 (17.1)
Northern Ontario resident	39 (14.2)	31 (12.0)	27 (11.5)	36 (14.8)	32 (14.3)	37 (14.7)	43 (18.0)^b^	25-29 (9.8-11.2)^b^	35-39 (13.6-14.9)	39 (14.8)	34 (14.1)	33 (12.8)
Lowest income quintile^c^	93 (33.8)	91 (35.1)	87 (37.0)	89 (36.6)	82 (36.8)	86 (34.1)	85 (35.6)	83-87 (32.5-33.6)	86-90 (33.5-34.5)	93 (35.2)	75 (31.1)	86 (33.3)
Long-term care resident	14 (5.1)^b^	28 (10.8)^b^	21 (8.9)	25 (10.3)	24 (10.8)	28 (11.1)	22 (9.2)	35-39 (13.7-15.1)	28-32 (10.9-12.3)	25 (9.5)	17 (7.1)	15 (5.8)
Congestive heart failure	99 (36.0)	101 (39.0)	83 (35.3)	98 (40.3)	84 (37.7)	107 (42.5)	87 (36.4)	110-114 (43.1-44.0)	97-101 (37.7-38.7)	100 (37.9)	65 (27.0)^b^	96 (37.2)^b^
Hypertension	244 (88.7)	222 (85.7)	193 (82.1)	210 (86.4)	192 (86.1)	218 (86.5)	198 (82.8)^b^	234-239 (91.8-92.3)^b^	217-221 (84.4-84.7)	233 (88.3)	205 (85.1)	226 (87.6)
COPD	93 (33.8)	87 (33.6)	71 (30.2)	88 (36.2)	79 (35.4)	93 (36.9)	82 (34.3)	97-101 (38.0-39.0)	83-87 (32.3-33.3)	92 (34.8)	61 (25.3)	78 (30.2)
Coronary artery disease	70 (25.5)	65 (25.1)	71 (30.2)	83 (34.2)	53 (23.8)	77 (30.6)	63 (26.4)	79-83 (31.0-32.0)	68-72 (26.5-27.6)	76 (28.8)	68 (28.2)	69 (26.7)
Chronic kidney disease	107 (38.9)	109 (42.1)	91 (38.7)	98 (40.3)	109 (48.9)^b^	87 (34.5)^b^	96 (40.2)	109-113 (42.7-43.6)	112-116 (43.6-44.4)	114 (43.2)	92 (38.2)	103 (39.9)
Peripheral artery disease	154 (56.0)^b^	173 (66.8)^b^	131 (55.7)	157 (64.6)	136 (61.0)	154 (61.1)	145 (60.7)	157-161 (61.6-62.2)	146-150 (56.8-57.5)	166 (62.9)	145 (60.2)	147 (57.0)

Abbreviation: COPD. chronic obstructive pulmonary disease.

^a^ In periods where 5 or fewer people experienced multiple major amputations within a given time period, a range in the number of amputees is provided rather than exact count in order to ensure patient privacy.

^b^ *P* < .05 for 2020-2021 vs 2019-2020 period.

^c^ Rural residence or income quintile missing in 0 to 5 individuals who underwent amputation per period (exact numbers suppressed for anonymity).

**Figure 1.**
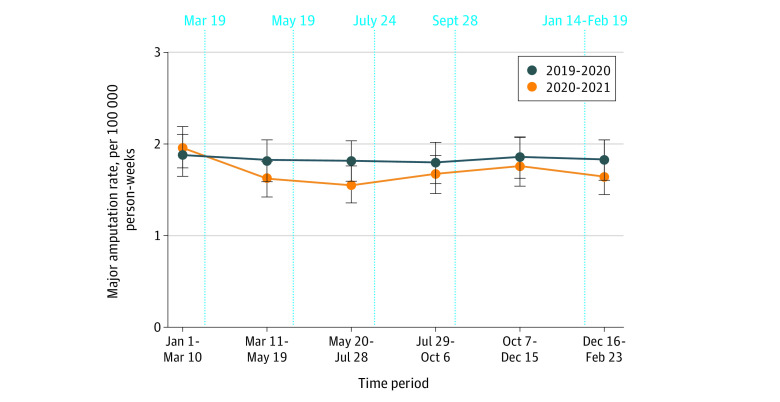
Major Amputation Rates Among Adults With Diabetes Major amputation indicates above-ankle amputations. On March 19, 2020, nonessential health services and scheduled procedures were suspended; May 19, 2020, gradual resumption in scheduled procedures and in-person health services permitted; July 24, 2020, provincial state of emergency lifted; September 28, 2020, official recognition of second wave; January 14, 2021 to February 19, 2021, second provincial state of emergency. Error bars indicate 95% CIs. Table 3 contains counts, rates, and rate ratios for 2020-2021 vs 2019-2020 per time period.

**Figure 2.**
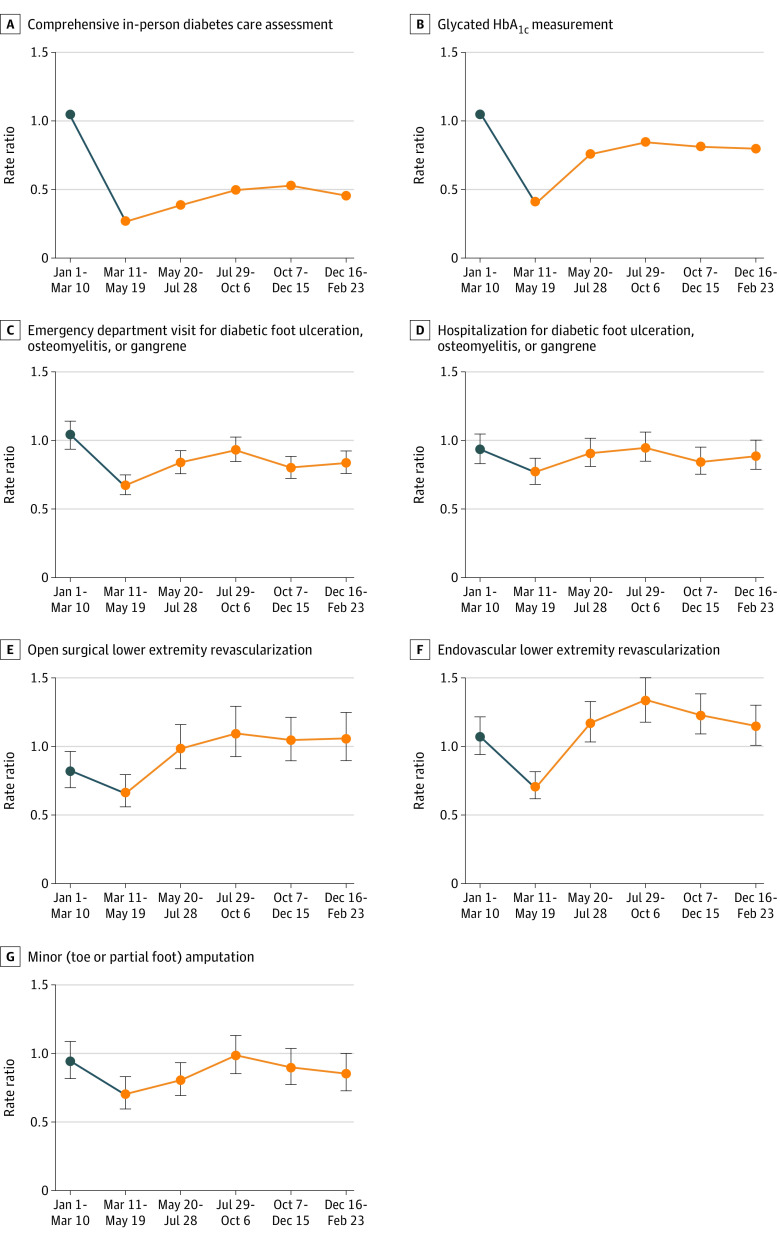
Outcome and Care Measure Rate Ratios for 2020-2021 Periods Relative to 2019-2020 Control Periods The blue dot indicates the rate ratio during the prepandemic period; orange dots, COVID-19 period, starting on March 11, 2020. C-G, Error bars indicate 95% CIs.

The rate of major amputation among people with diabetes in 2020-2021 never exceeded 2019-2020 rates. In fact, as the pandemic progressed, amputation rates decreased twice, reaching a nadir at 86% of 2019-2020 levels during the latter half of the first wave (May 20 to July 28, 2019 rate: 1.81; May 20 to July 28, 2020 rate: 1.55) (Figure 1 and Table 3). The RR for the prepandemic period from January 1 to March 10 was 1.05 (95% CI, 0.88-1.25), with RRs in the pandemic period ranging from 0.86 (95% CI, 0.72-1.03) in May 20 to July 28 to 0.95 (95% CI, 0.80-1.13) in October 7 to December 15. In keeping with scaling back in-person physician assessments, the rate of comprehensive in-person diabetes care assessment immediately dropped to 28% of the 2019 level (RR, 0.28; 95% CI, 0.28-0.28) in March 11 to May 19 (Figure 2 and Table 3; eAppendix 2 in the Supplement). These remained low over the study period. The rate of HbA_1c_ measurement also initially dropped to 41% of the 2019 level (RR, 0.41; 95% CI, 0.40-0.41) but increased to 84% between the first and second waves (RR, 0.84; 95% CI, 0.84-0.85) (Figure 2 and Table 3). ED visits and hospitalizations for diabetic foot ulceration, osteomyelitis or gangrene declined early in the pandemic (ED visits, March 11 to May 19: RR, 0.67; 95% CI, 0.61-0.75; hospitalizations, March 11 to May 19: RR, 0.77; 95% CI, 0.68-0.87); however, there was recovery to 2019 levels in the period of July 29 to October 6 (Figure 2 and Table 3). Rates of lower extremity revascularization and minor amputation rebounded after an initial pandemic-related first-wave decline (open revascularization, March 11 to May 19: RR, 0.66; 95% CI, 0.56-0.79; endovascular revascularization, March 11 to May 19: RR, 0.70; 95% CI, 0.61-0.81; minor amputation, March 11 to May 19: RR, 0.70; 95% CI, 0.60-0.83) (Figure 2 and Table 3).

**Table 3.** Outcomes and Care Measures Among Adults With Diabetes by Time Period

Indicator measures^a^	Time period							
	Jan 1-Mar 10 (pre–COVID-19)	Wave 1				Jul 29-Oct 6 (between waves)	Wave 2	
		Mar 11–May 19		May 20-Jul 28			Oct 7-Dec 15	Dec 16-Feb 23
**Major (above-ankle) amputations**								
2020-2021, No.	288		241		230	248	262	247
2019-2020 Control period, No.	267		260		262	260	270	268
Rate in 2020-2021, per 100 000 person-weeks	1.96		1.63		1.55	1.67	1.75	1.64
Rate in 2019-2020 control period, per 100 000 person-weeks	1.87		1.81		1.81	1.79	1.85	1.82
Rate ratio (95% CI)	1.05 (0.88-1.25)		0.90 (0.75-1.08)		0.86 (0.72-1.03)	0.93 (0.78-1.12)	0.95 (0.80-1.13)	0.90 (0.76-1.08)
**Comprehensive in-person diabetes care assessments**								
2020-2021, No.	339 997	99 781		131 069		168 048	195 511	145 844
2019-2020 Control period, No.	312 901	342 898		329 277		326 964	358 759	307 698
Rate in 2020-2021, per 100 000 person-weeks	2314.11	674.35		884.04		1130.49	1307.52	970.10
Rate in 2019-2020 control period, per 100 000 person-weeks	2197.12	2393.05		2280.23		2251.89	2455.28	2092.85
Rate ratio (95% CI)	1.05 (1.04-1.05)	0.28 (0.28-0.28)		0.39 (0.38-0.39)		0.50 (0.50-0.50)	0.53 (0.53-0.53)	0.46 (0.46-0.46)
**Hemoglobin A_1c_ measurements**								
2020-2021, No.	538 491	221 195		402 442		452 117	451 950	383 790
2019-2020 Control period, No.	499 258	525 681		515 867		522 672	541 738	472 363
Rate in 2020-2021, per 100 000 person-weeks	3665.12	1494.91		2714.41		3041.49	3022.51	2552.83
Rate in 2019-2020 control period, per 100 000 person-weeks	3505.67	3668.68		3572.36		3599.79	3707.56	3212.85
Rate ratio (95% CI)	1.04 (1.04-1.05)	0.41 (0.40-0.41)		0.76 (0.76-0.76)		0.84 (0.84-0.85)	0.82 (0.81-0.82)	0.79 (0.79-0.80)
**Emergency department visits for diabetic foot ulceration, osteomyelitis, or gangrene**								
2020-2021, No.	1105	781		1044		1169	940	956
2019-2020 Control period, No.	1045	1130		1224		1238	1161	1126
Rate in 2020-2021, per 100 000 person-weeks	7.52	5.28		7.04		7.86	6.29	6.36
Rate in 2019-2020 control period, per 100 000 person-weeks	7.34	7.89		8.48		8.53	7.95	7.66
Rate ratio (95% CI)	1.03 (0.93-1.14)	0.67 (0.61-0.75)		0.84 (0.76-0.92)		0.93 (0.84-1.02)	0.79 (0.72-0.88)	0.83 (0.75-0.92)
**Hospitalizations for diabetic foot ulceration, osteomyelitis, or gangrene**								
2020-2021, No.	670	534		638		664	604	582
2019-2020 control period, No.	704	677		695		694	706	646
Rate in 2020-2021, per 100 000 person-weeks	4.56	3.61		4.30		4.47	4.04	3.87
Rate in 2019-2020 control period, per 100 000 person-weeks	4.94	4.72		4.81		4.78	4.83	4.39
Rate ratio (95% CI)	0.93 (0.83-1.04)	0.77 (0.68-0.87)		0.90 (0.80-1.01)		0.94 (0.84-1.06)	0.84 (0.75-0.94)	0.89 (0.79-1.00)
**Open surgical lower extremity revascularizations**								
2020-2021, No.	284	217		298		298	354	282
2019-2020 Control period, No.	337	318		295		267	333	262
Rate in 2020-2021, per 100 000 person-weeks	1.93	1.47		2.01		2.00	2.37	1.88
Rate in 2019-2020 control period, per 100 000 person-weeks	2.37	2.22		2.04		1.84	2.28	1.78
Rate ratio (95% CI)	0.82 (0.70-0.96)	0.66 (0.56-0.79)		0.98 (0.84-1.16)		1.09 (0.92-1.29)	1.04 (0.90-1.21)	1.06 (0.89-1.25)
**Endovascular lower extremity revascularizations**								
2020-2021, No.	505	347		551		576	595	532
2019-2020 Control period, No.	463	479		463		425	478	457
Rate in 2020-2021, per 100 000 person-weeks	3.44	2.35		3.72		3.87	3.98	3.54
Rate in 2019-2020 control period, per 100 000 person-weeks	3.25	3.34		3.21		2.93	3.27	3.11
Rate ratio (95% CI)	1.06 (0.94-1.21)	0.70 (0.61-0.81)		1.16 (1.03-1.32)		1.33 (1.17-1.51)	1.22 (1.08-1.38)	1.14 (1.01-1.30)
**Minor (toe or partial-foot) amputations**								
2020-2021, No.	359	260		325		388	371	296
2019-2020 Control period, No.	372	359		395		387	406	342
Rate in 2020-2021, per 100 000 person-weeks	2.44	1.76		2.19		2.61	2.48	1.97
Rate in 2019-2020 control period, per 100 000 person-weeks	2.61	2.51		2.74		2.67	2.78	2.33
Rate ratio (95% CI)	0.94 (0.81-1.09)	0.70 (0.60-0.83)		0.80 (0.69-0.93)		0.98 (0.85-1.13)	0.89 (0.77-1.03)	0.85 (0.72-0.99)

^a^ Counts of adults with diabetes and total person-weeks per time period presented in eAppendix 2 in the Supplement.

The 50-week cumulative incidences of major amputation and death without major amputation among adults with diabetes in the March 11, 2020, cohort of 1 488 605 individuals were 0.08% (1143 patients) and 3.07% (45 689 patients), respectively. The cumulative incidences of major amputation and death without major amputation among 1 441 029 adults with diabetes in the March 11, 2019, cohort were 0.09% (1220 patients) and 2.77% (39 985 patients), respectively.

## Discussion

Our population-based analysis found that adults living with diabetes in Ontario did not undergo more amputations during the first 2 waves of the COVID-19 pandemic compared with historical data, despite limited ambulatory in-person assessment by physicians, hospital avoidance, and restrictions to scheduled hospital-based procedures. These results are in stark contrast to concern fueled by dire reports from other jurisdictions. In central China, a multidisciplinary diabetic foot care team reported that 11.4% of their patients hospitalized with a diabetic foot ulcer (DFU) required major amputation during the pandemic compared with 4.6% in historical controls of similar comorbidity level.^5^ In Naples, Italy, minor amputations increased by a factor of 2.5 with the onset of the pandemic among hospitalized patients with DFU.^6^ In Ohio, a study of foot and ankle service consultations identified a spike in those requiring major amputations from 6.9% in the first 3 months of 2020 to 18% in the subsequent 5 months, driven by greater foot infection severity.^7^ Vascular surgeons in the Netherlands analyzed their operative cases and identified an increase in the proportion of major amputations (42% in the 2020 pandemic period compared with 18% in the corresponding 2019 period), attributed to more extensive tissue ischemia.^8^ Similarly, in a hospital in Chennai, India, the number of major amputations increased by 54% in March to December 2020 compared with a similar period in 2019.^9^ Notwithstanding the limitations of these studies, there are a number of explanations for more positive results to be observed in Ontario. First, hospital resources were not overburdened by patients with COVID-19 in the first wave of the pandemic. As a result, hospital care restrictions in Ontario were severe but relatively brief (approximately 10 weeks) and were not reinstated during the second wave. Second, invasive procedures necessary to treat limb-threatening complications of diabetes, such as toe or partial-foot amputations and revascularization, remained prioritized during care restrictions.^22^ Third, in keeping with other jurisdictions,^23^ a shift to virtual visits may have supported resumption of some screening tests, as evidenced by the trend in HbA_1c_ measurement. Fourth, the importance of maintaining capacity to evaluate urgent ambulatory presentations in person was widely recognized, including by wound care and surgical specialists.^22^ Fifth, it may be that societal lockdowns in the first and second waves contributed to a relative reduction in daily activities among people with diabetes at greatest risk of foot ulceration. A lower activity level would have resulted in less repetitive microtrauma to the foot and toes, a key initiating factor in the development of diabetic foot ulceration in the presence of neuropathy. This hypothesis supports the value of proper footwear and offloading of pressure points and is further supported by recent studies internationally. Lipscomb et al,^24^ working within a regional ambulatory diabetes care program in the United Kingdom, reported fewer DFU events observed during their Spring 2020 lockdown. Mariet and colleagues^11^ reported fewer DFUs and fewer amputations during the 2020 lockdown across France. Valabhji et al^12^ also advanced this hypothesis in their report showing fewer amputations among people with diabetes in England. However, they also raised the possibility that excess mortality related to COVID-19 may have acted as a competing risk to amputation.^12^ Our longitudinal analysis showed an increase in mortality in the March 11, 2020, vs March 11, 2019, diabetes cohorts consistent with a hypothesis that excess pandemic-related deaths may have reduced total amputations. However, we also found that the proportion of people living in long-term care settings, in whom approximately 80% of COVID-19–related first-wave deaths occurred,^25^ was not lower among individuals undergoing amputation in the 2020-2021 period compared with those in the 2019-2020 period. Furthermore, the comorbidity level among individuals undergoing amputation in 2020-2021 relative to the 2019-2020 control groups was not consistently lower with the onset of the pandemic. Thus, the demographic and comorbidity profiles of individual undergoing amputation during the pandemic, when compared with the 2019-2020 control groups, do not suggest that older adults with limb-threatening complications died during the pandemic rather than underwent amputation. Nevertheless, at an individual level, it remains possible that some deaths during the pandemic occurred in patients who might otherwise have undergone amputation. More granular data on the foot health of decedents with diabetes during the pandemic are necessary to further clarify this issue.

The result of our analysis and those in England and France can be viewed as a positive outcome as governments sought to maintain essential health services during a time of unprecedented uncertainty. However, as we emerge from a third severe wave of COVID-19 and some jurisdictions are facing additional waves, we must remain vigilant about the potential long-term consequences of the pandemic on the risk of diabetic foot complications and limb loss. Detrimental associations with risk factor modification (eg, income disruption limiting access to medications or periodic podiatry assessment, psychological stress exacerbating unhealthy behaviors such as smoking or poor diet) may not be immediately apparent. Going forward, multipronged prevention of diabetic foot complications—including periodic foot screening and footwear evaluation, glycemia management, cardiovascular and kidney disease risk factor management, and patient education—must be prioritized while strengthening the capacity to salvage a threatened limb through accessible multidisciplinary limb preservation expertise. The silver lining of the pandemic for diabetic foot care may also turn out to be the acceleration of novel applications of virtual care (eg, for remote wound monitoring) and wearable technologies (eg, for self-assessment of pressure offloading) that can support improvements in the prevention and treatment of diabetic foot complications.^26,27^

### Strengths and Limitations

This analysis has important strengths. These include a population-based scope supported by validated identification of people with type 1 or 2 diabetes, the inclusion of diverse care measures and outcomes related to diabetic foot complications, and 11 months of complete data during the pandemic.

Certain limitations of this analysis also warrant emphasis. First, we cannot reliably identify ambulatory clinic care for diabetic foot ulceration or gangrene, nor can we directly quantify the severity of diabetic foot ulceration or gangrene at the time of hospital presentation. However, the most severe cases would be captured by ED visits, hospitalization, and/or amputation. Second, although the coding of lower extremity revascularization has been validated, the diagnostic coding of diabetic foot complications in ED and hospitalization records is of uncertain validity. There is, however, no reason to expect that diagnostic coding differences exist between 2020 and 2019. Third, our analysis compares overlapping cohorts of all adults with diabetes in 2019-2020 vs 2020-2021 periods. Therefore, secular trends (eg, ongoing decrease in event rates from 2019 through 2021) may influence the observed results. However, if the decrease in rates seen with the onset of the pandemic on March 11, 2020, was strongly associated with secular trends, we would expect to also see lower rates in January 1 to March 10, 2020 (the 2020 prepandemic period), vs January 1 to March 10, 2019. With the exception of open revascularization, all rates in the 2020 prepandemic period were similar to 2019 rates (Table 3 and Figure 2). Therefore, it seems more likely pandemic-related events, rather than secular trends, explain the decrease in rates seen after March 11, 2020. Fourth, the time period of the analysis included the full first and second waves but not the third wave of the pandemic, so the results cannot be considered to estimate the experience in the early summer of 2021.

## Conclusions

In conclusion, this analysis found that the COVID-19 pandemic was not associated with additional limb loss for people living with diabetes. However, as we emerge from the pandemic, ongoing efforts to strengthen comprehensive diabetes care, including foot screening as well as improving access to interdisciplinary limb salvage expertise, remain critical to maintain these positive results.

## Notes

Supplement. **eAppendix 1. **Database, Coding Definitions, and Supporting References **eAppendix 2. **Count of Adults with Diabetes and Total Person-Weeks per Time Period
