# Supplementary material for: A Population-Based Analysis of Diabetes-Related Care Measures, Foot Complications, and Amputation During the COVID-19 Pandemic in Ontario, Canada
Source: JAMA Netw Open. 2022 Jan 5;5(1):e2142354. doi: 10.1001/jamanetworkopen.2021.42354 (PMC8733837; doi:10.1001/jamanetworkopen.2021.42354)
Supplement: Supplement. — eAppendix 1. Database, Coding Definitions, and Supporting References eAppendix 2. Count of Adults with Diabetes and Total Person-Weeks per Time Period [file jamanetwopen-e2142354-s001.pdf]

Supplemental Online Content

de Mestral C, Gomez D, Wilton AS, et al. A population-based analysis of diabetes-related care measures, foot complications, and amputation during the COVID-19 pandemic in Ontario, Canada. *JAMA Netw Open*. 2022;5(1):e2142354. doi:10.1001/jamanetworkopen.2021.42354

eAppendix 1. Database, Coding Definitions, and Supporting References

eAppendix 2. Count of Adults with Diabetes and Total Person-Weeks per Time Period

This supplemental material has been provided by the authors to give readers additional information about their work.

© 2022 de Mestral C et al. *JAMA Network Open.*

eAppendix 1. Database, Coding Definitions, and Supporting References

Population-based Databases	Description
Registered Persons Database (RPDB)	Records of demographics and vital statistics for all Ontario Residents [1]
Discharge Abstract Database (DAD)	Records of all hospitalizations in Ontario acute care hospitals [1]
Same Day Surgery (SDS)	Records of all same day surgeries and invasive procedures in Ontario [1]
Ontario Health Insurance Plan Claims Database (OHIP)	Records of all physician service claims in Ontario [1]
National Ambulatory Care Reporting System (NACRS)	Records of all emergency department visits in Ontario [1]
Continuing Care Reporting System Database	Records of all admissions to publicly-funded long-term care (a.k.a. nursing home) facilities in Ontario [1]
Ontario Diabetes Database	Disease-specific ICES-derived cohort [1,2]
Ontario Hypertension Database	Disease-specific ICES-derived cohort [1,3]
Ontario Congestive Heart Failure Database	Disease-specific ICES-derived cohort [1,4]
Ontario Chronic Obstructive Pulmonary Disease Database	Disease-specific ICES-derived cohort [1,5]

© 2022 de Mestral C et al. *JAMA Network Open.*

eAppendix 1. Database, Coding Definitions, and Supporting References (continued)

Diagnosis, Measure or Intervention	Database	Codes	Supporting References
Diabetes Mellitus (Type 1 or 2)	Ontario Diabetes Database	Diagnosis date that precedes the index time period	2
Major Amputation	DAD	CCI: 1VC93, 1VG93, 1VQ93	6
Comprehensive Diabetes Management Assessment	OHIP	Physician Service Fee:<br>Primary care: K030, Q040; Endocrinologist or general internal medicine specialist: K045, K046	7, 8
Glycated Hemoglobin (A1c) Measurement	OHIP	Laboratory Service Fee:<br>L093	9
Diabetic Foot Ulcer, Osteomyelitis or Gangrene	DAD<br>NACRS	ICD-10:<br>Diabetic foot ulcer with or without gangrene: E10.51, E10.70, E10.71, E11.51, E11.70, E11.71, E13.51, E13.70, E13.71, E14.51, E14.70, E14.71<br>Osteomyelitis of the foot: M86.17, M86.27, M86.37, M86.47, M86.67, M86.87, M86.97<br>Atherosclerosis of lower extremities with gangrene: I70.21	10
Open Surgical Lower Extremity Revascularization	DAD<br>SDS	CCI: 1KA57LA, 1KA76MZ, 1KE57LA, 1KE76MZ, 1KG57LA, 1KG76MI, 1KT57LA, 1KT76MZ, 1JM76MI	11
Endovascular Lower Extremity Revascularization	DAD<br>SDS	CCI: 1KA50GQ-BD, 1KA57GQ, 1KE35, 1KE50GQ, 1KE57GQ, 1KG35, 1KG50GQ, 1KG57GQ, 1KT50GQ, 1KT57GQ	11
Minor Amputation	DAD<br>SDS	CCI: 1WA93, 1WE93, 1WI93, 1WJ93, 1WK93, 1WL93, 1WM93, 1WN93	6
**Patient Characteristics**			
Age & Sex	RPDB	-	-
Neighbourhood of residence Income Quintile, Rurality and Northern Region – LHINs 13 and 14	RPDB	-	12,13
Long-term Care Residence	CCRS	Admitted to long-term care facility at beginning of given time period	14
Hypertension	Ontario Hypertension Database	Diagnosis date that precedes the index time period	3
Congestive Heart Failure	Ontario Heart Failure Database	Diagnosis date that precedes the index time period	4
Chronic Obstructive Pulmonary Disease	Ontario COPD Database	Diagnosis date that precedes the index time period	5

© 2022 de Mestral C et al. *JAMA Network Open.*

Coronary Artery Disease	DAD (5-year lookback)	<u>ICD-10:</u><br>I20, I21, I22, I23, I24, I25	15
Chronic Kidney Disease	DAD (5-year lookback)	<u>ICD-10:</u><br>I12, I13, N08.3, N18, E10.2, E11.2, E13.2, E14.2	16
Peripheral Artery Disease	DAD (5-year lookback)	<u>ICD-10:</u><br>I70.0, I70.2, I70.8, I70.9, I73.9, I74.0, I74.3, I74.4, I74.5, I74.8, I74.9, I79.2, E10.50, E10.51, E10.70, E10.71, E11.50, E11.51, E11.70, E11.71, E13.50, E13.51, E13.70, E13.71, E14.50, E14.51, E14.70, E14.71	6,11

Abbreviations: ICD-10-CA, International Statistical Classification of Diseases, Tenth Revision, Canadian Version; CCI, Canadian Classification of Health Interventions; DAD, Discharge Abstract Database; SDS, Same Day Surgery; OHIP, Ontario Health Insurance Plan*;* NACRS, National Ambulatory Care Reporting System; RPDB, Registered Persons Database; CCRS, Continuing Care Reporting System; LHIN – Local Health Integrated Network

© 2022 de Mestral C et al. *JAMA Network Open.*

eAppendix 1 References:

1. ICES Data Dictionary. https://www.ices.on.ca/Data-and-Privacy/ICES-data/Data-dictionary (accessed June 7, 2021)
2. Hux JE, Ivis F, Flintoft V, Bica A. Diabetes in Ontario: determination of prevalence and incidence using a validated administrative data algorithm. Diabetes Care. 2002; 25:512–516
3. Tu K, Chen Z, Lipscombe LL; Canadian Hypertension Education Program Outcomes Research Taskforce. Prevalence and incidence of hypertension from 1995 to 2005: a population-based study. CMAJ. 2008;178(11):1429-1435.
4. Schultz SE, Rothwell DM, Chen Z, Tu K. Identifying cases of congestive heart failure from administrative data: a validation study using primary care patient records. Chronic Dis Inj Can. 2013;33(3):160-166.
5. Gershon AS, Wang C, Guan J, Vasilevska-Ristovska J, Cicutto L, To T. Identifying individuals with physcian diagnosed COPD in health administrative databases. COPD. 2009;6(5):388-394.
6. Hussain MA, Al-Omran M, Salata K, Sivaswamy A, Forbes TL, Sattar N, Aljabri B, Kayssi A, Verma S, de Mestral C. Population-Based Secular Trends in Lower Extremity Amputation for Diabetes and Peripheral Artery Disease. CMAJ 2019:191(35): E655
7. Kiran T, Victor JC, Kopp A, Shah BR, Glazier RH. The relationship between financial incentives and quality of diabetes care in Ontario, Canada. Diabetes Care. 2012 May;35(5):1038-46.
8. Ontario Health Insurance Plan. Schedule of Benefits for Physician Services – Schedule of Benefits. https://www.health.gov.on.ca/en/pro/programs/ohip/sob/physserv/sob_master20210314.pdf
9. Ontario Health Insurance Plan. Schedule of Benefits for Laboratory Services. https://www.health.gov.on.ca/en/pro/programs/ohip/sob/lab/lab_mn2020.pdf
10. Hopkins RB, Burke N, Harlock J, Jegathisawaran J, Goeree R. Economic burden of illness associated with diabetic foot ulcers in Canada. BMC Health Serv Res. 2015;15(1):13.
11. Jacob-Brassard J, Al-Omran M, Stukle TA, Mamdani M, Lee DS, de Mestral C. Validation of diagnosis and procedure codes for revascularization for peripheral artery disease in Ontario Administrative Databases. Clin Invest Med 2021; 44(2):E36-43
12. Wilkins R. Use of postal codes and addresses in the analysis of health data. Health Rep 1993;5:157-77.
13. Kralj B. Measuring rurality — RIO2008_BASIC: methodology and results. Toronto: Ontario Medical Association; 2009.
14. Lane NE, Wodchis WP, Boyd CM, Stukel TA. Disability in long-term care residents explained by prevalent geriatric syndromes, not long-term care home characteristics: a cross-sectional study. BMC Geriatr. 2017; 17:49.
15. Tu JV, Chu A, Donovan LR, et al. The Cardiovascular Health in Ambulatory Care Research Team (CANHEART): using big data to measure and improve cardiovascular health and healthcare services. Circ Cardiovasc Qual Outcomes. 2015;8(2):204-212.
16. Fleet JL, Dixon SN, Shariff SZ, et al. Detecting chronic kidney disease in population-based administrative databases using an algorithm of hospital encounter and physician claim codes. BMC Nephrol. 2013;14:81.

© 2022 de Mestral C et al. *JAMA Network Open.*

eAppendix 2. Count of Adults with Diabetes and Total Person-Weeks per Time Period

	TIME PERIODS					
	**Jan 1-Mar 10**	**Mar 11-May 19**	**May 20-Jul 28**	**Jul 29-Oct 6**	**Oct 7-Dec 15**	**Dec 16-Feb 23**
	Pre-COVID-19	Wave 1	Wave 1	Between Waves	Wave 2	Wave 2
**No. of adults with diabetes** – 2020/21	1,478,808	1,488,605	1,489,950	1,496,050	1,504,105	1,512,312
**No. of adults with diabetes** – 2019/20 Control Periods	1,432,934	1,441,029	1,451,135	1,461,236	1,469,824	1,478,513
**Total Person-weeks** – 2020/21	14,692,328	14,796,514	14,826,149	14,864,998	14,952,812	15,033,917
**Total Person-weeks** – 2019/20 Control Periods	14,241,437	14,328,900	14,440,523	14,519,515	14,611,727	14,702,328

© 2022 de Mestral C et al. *JAMA Network Open.*
